# Maternal atopic conditions and autism spectrum disorder: a systematic review

**DOI:** 10.1007/s00787-023-02285-7

**Published:** 2023-09-03

**Authors:** Asilay Seker, Anxhela Qirko-Gurakuqi, Mirela Tabaku, Kenneth Ross P. Javate, Iris Rathwell

**Affiliations:** 1 Institute of Psychiatry, Psychology and Neuroscience, King’s College London, London, UK; 2 South London and Maudsley NHS Foundation Trust, London, UK; 3 Department of Biomedical and Experimental Subjects, University of Medicine, Tirana, Albania; 4 Paediatric Department, University of Medicine, Tirana, Albania; 5 Department of Psychiatry, The Medical City Hospital, Manila, Philippines; 6 School of Medicine and Public Health, Ateneo de Manila University, Manila, Philippines

**Keywords:** Atopy, Autism, Maternal, ASD, Immune activation

## Abstract

Autism spectrum disorder (ASD) is a disabling neurodevelopmental condition with complex etiology. Emerging evidence has pointed to maternal atopy as a possible risk factor. It is hypothesized that maternal atopic disease during pregnancy can lead to increased levels of inflammatory cytokines in fetal circulation via placental transfer or increased production. These cytokines can then pass through the immature blood–brain barrier, causing aberrant neurodevelopment via mechanisms including premature microglial activation. The objective of this study is to systematically review observational studies that investigate whether a maternal history of atopic disease (asthma, allergy, or eczema/atopic dermatitis) is associated with a diagnosis of ASD in offspring. A search was conducted in Ovid MEDLINE, PsycINFO, and Embase databases for relevant articles up to November 2021; this was later updated in January 2022. Observational studies published in peer-reviewed journals were included. Data were synthesized and qualitatively analyzed according to the specific atopic condition. Quality assessment was done using the Newcastle–Ottawa Scale. Nine articles were identified, with all including asthma as an exposure, alongside four each for allergy and eczema. Findings were inconsistent regarding the association between a maternal diagnosis of either asthma, allergy, or eczema, and ASD in offspring, with variations in methodology contributing to the inconclusiveness. More consistent associations were demonstrated regarding maternal asthma that was treated or diagnosed during pregnancy. Evidence suggests that symptomatic maternal asthma during pregnancy could be associated with ASD in offspring, underscoring the importance of effective management of atopic conditions during pregnancy. Further research is needed, particularly longitudinal studies that use gold-standard assessment tools and correlate clinical outcomes with laboratory and treatment data.

*PROSPERO Registration Number and Date*: CRD42018116656, 26.11.2018.

## Introduction

Autism Spectrum Disorder (ASD) is a neurodevelopmental condition distinguished by early-onset deficits in social communication, as well as restricted, repetitive behaviors and interests [1, 2]. This disorder may cause significant impairment in daily functioning, and is associated with high levels of comorbidity with other medical, developmental, and psychiatric conditions [3]. Prevalence rates have risen over the past two decades, with the median worldwide prevalence estimated at 0.62–0.7% [4, 5]. Despite great strides in awareness and understanding, much is still not known regarding the etiology of ASD. Current evidence points to autism as resulting from the interaction of genetic factors with the environment, both prenatal and postnatal [6, 7].

Recent studies propose inflammation as a notable risk factor [8], with specific attention to maternal immune dysregulation during pregnancy potentially affecting vulnerable stages of fetal brain development [9, 10]. Pre-clinical studies have demonstrated that maternal immune activation in mice and rhesus monkeys led to increased autism-like behaviors in offspring [11, 12]. Recent reviews and meta-analyses have also concluded that ASD in offspring is associated with both maternal autoimmune disease [13] and maternal infection [14, 15] which further indicates the potential role of maternal immune activation (MIA) on fetal neurodevelopment.

Emerging evidence has also pointed to maternal atopy, a distinct form of immune dysregulation, as a risk factor. In 2005, a case–control study initially reported an association between a diagnosis of maternal asthma and allergy with autism in offspring [16], setting the stage for other recent studies to investigate the potential relationship [15, 17, 18]. It is hypothesized that maternal atopic disease during pregnancy can lead to increased levels of inflammatory cytokines in fetal circulation, either through placental transfer or increased production. These cytokines can then pass through the immature blood–brain barrier, causing premature microglial activation and consequently, aberrant neurodevelopment [9, 19, 20].

In line with the above hypothesis regarding atopy, a recent cohort study concludes that the maternal prenatal IgE, an immunoglobulin with an essential role in atopic reactions, may influence neurodevelopment of the offspring [21, 22].

Despite the links between MIA and ASD pathophysiology, some important concerns how MIA impacts the developing fetal brain at genomic and epigenomic levels need still to be clarified [23]^.^ Autism Spectrum Disorder and other neurodevelopmental diseases in humans are caused by the vulnerability of developing brain to epigenetic modifications, which are assumed to be the genome's adaptation to a changing environment [24–26]. Research studies suggest that epigenetic mechanisms may play a role in the relationship between environmental factors and immune system changes [27]. The changes in the expression and methylation of immune system-related genes in ASD could happen in response to environmental factors such as MIA [24].

Some studies on animal models reveal that offspring's long-term behavioral abnormalities after exposure to prenatal maternal allergic asthma (MAA) are due to epigenetic modification within the brain cells, via DNA methylation and histone acetylation [24, 28, 29]. Vogel et al. performed first whole genome-wide analyses of the DNA methylome and transcriptome of microglia isolated from juvenile offspring of MAA dams (mother of an animal) and their findings suggest an overlap between MAA and ASD-relevant epigenetic changes in microglia [24]. Another study by Lombardo et al. reported a potential relationship between ASD risk and dysregulation in cerebral gene expression in fetus as a result of MIA [23].

Another recent Swedish population case–control study found association between maternal asthma and increased risk of offspring ASD [17]. Although there are reports of mixed evidence on the associations between maternal asthma during pregnancy and child cognitive and behavioral development [30], one of the most recent systematic reviews [15] found that exposure to maternal asthma in the first and second trimester is associated with childhood ASD, which has consolidated the evidence regarding MIA as one of the pathways causing fetal neuroinflammation.

It is hoped that the current review will contribute to fill the knowledge gap in the understanding of inflammation-related prenatal risk factors for ASD and complement the existing reviews which investigate atopic diseases in children and their risk for autism [31–33].

Potentially, this review also has clinically relevant implications in terms of treatment and management of maternal atopic conditions towards healthy outcomes in neurodevelopmental disorders [34].

Therefore, the objective of this study is to systematically review cross-sectional, case–control, and cohort studies that investigate whether a maternal history of atopic disease (asthma, allergy, or eczema/atopic dermatitis) is associated with a diagnosis of autism spectrum disorder in offspring.

## Methodology

### Literature search

This systematic review was conducted according to the recommendations of the Preferred Reporting Items for Systematic Reviews (PRISMA) statement [35] and registered with PROSPERO (CRD42018116656).

A search was conducted in Ovid MEDLINE, PsycINFO, and Embase databases for articles up to January 2022, without restrictions regarding language, date, or article type. Keywords used were maternal OR prenatal OR pregnancy OR famil* AND atopy OR asthma OR allerg* OR eczema OR atopic dermatitis AND autis* OR Asperger OR ASD.

### Study selection

Titles and abstracts of articles were screened to exclude discernibly irrelevant studies, after which the full text of the remaining studies were assessed according to inclusion criteria. In addition, the reference lists of the included articles were examined, and further articles were identified this way to be included in the review.

Observational studies that investigated the association between maternal history of atopic disease (asthma, allergy, or eczema) and autism spectrum disorder in offspring were included. Studies were excluded if they were case reports, case series, or animal studies. Studies that were not published in peer-reviewed journals were likewise excluded.

### Data extraction and synthesis

The following data were obtained from each study: author, publication year, country of origin, type of study, number of subjects, age, method of assessment of atopy and autism, and relevant study outcomes. Data were synthesized and qualitatively analyzed according to the specific atopic condition.

### Quality assessment

The Newcastle–Ottawa Scale was used to assess the risk of bias, as it was specifically developed to provide a useful means to assess the quality of non-randomized studies in systematic reviews [36]. In the scale, stars are awarded for quality characteristics grouped according to three categories: selection of study groups, comparability of study groups, and ascertaining outcome/exposure (for cohort and case–control studies, respectively), for which a total of nine stars may be awarded to indicate high quality.

Methodology involved multiple reviewers for the systematic search and data extraction, consensus was achieved through discussion where there were initial disagreements.

## Results

### Study selection

**Fig. 1:**
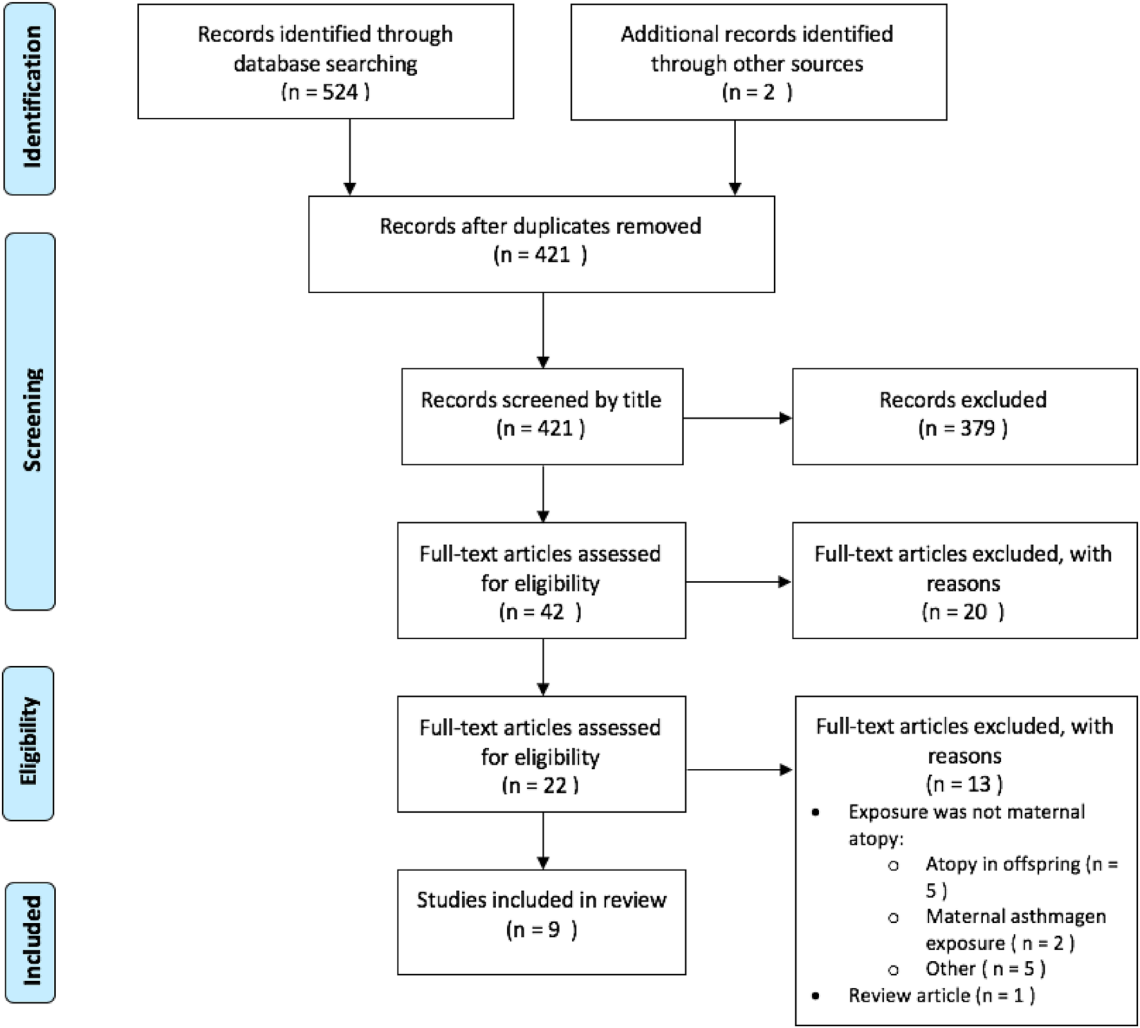
Flow diagram

The initial database search yielded 524 citations, with 122 from MEDLINE, 78 from PsycINFO, and 324 from Embase. An additional two articles were identified by reviewing reference lists. After removing duplicates, 421 studies were screened initially by title, yielding 42 studies, and then by abstract, yielding 22 studies. Of the studies which qualified for full-text screening, nine met the full inclusion criteria (see Fig. 1).

Study characteristics are summarized in Tables 1, 2 and 3. All of the nine studies included maternal asthma as an exposure variable, while four studies each included maternal allergy and eczema. All of the studies were conducted in high-income countries [37]: four in the USA, three in Australia, and one each in the UK and Denmark. Eight were case–control studies and one was a cohort study. Quality assessment using the Newcastle–Ottawa Scale is summarized in Table 4.

**Table 1:** Studies investigating maternal asthma and autism in offspring

References	Country	Type of study	Number of subjects	Age	Method of assessment of maternal asthma	Method of assessment of autism	Study outcomes (95% CI)
Croen et al. [16]	USA	Case–control	2502 (407/2095)	3–7	Recorded Physician Diagnosis (ICD-9; within 2 years of delivery)	Recorded Physician Diagnosis (ICD-9)	Asthma: 1.6 (1.2–2.2)
							Previous History: 1.2 (1.2–2.4)
							Trimester 1: 2.8 (1.3–6.1)
							Trimester 2: 2.2 (1.1–4.2)
							Trimester 3: 1.7 (1.0–2.8)
							Postnatal: 1.4 (0.8–2.3)
Croen et al. [40]	USA	Case–control	1647 (663/984)	2–5	Computer assisted telephone interview or Recorded Physician Diagnosis	SCQ if ≥ 11 then ADOS and ADI-R	Asthma: 1.26 (0.99–1.60)
							Asthma Treated During Pregnancy
							1.41 (1.07–1.85)
Hisle-Gorman et al. [38]	USA	Case–control	35,040 (8760/26,280)	2–18	Recorded Physician Diagnosis (ICD-9; Lifetime)/Prescription Record	Recorded Physician Diagnosis (ICD-9)	Diagnosis: 1.14 (1.01–1.29)
							Diagnosis and Prescription: 1.49 (1.29–1.74)
							Prescription: 1.29 (1.12–1.48)
Langridge et al. [39]	Australia	Case–control	383,153 (1179/376,529)	0–6	Prenatal Medical Record (Lifetime)	Recorded Physician Diagnosis (DSM IIIR-IV-TR)	Asthma + ASD with ID: 0.98 (0.71–1.36)
							Asthma + ASD without ID: 1.41 (0.98–2.04)
Leonard et al. [44]	Australia	Case–Control	237,155 (236,964/191)	0–16	Prenatal Medical Record (Lifetime)	Medical Record	Asthma + ASD with ID: 1.32 (0.65–2.67)
Lyall et al. [41]	USA	Case–control	951 (560/391)	2–5	Either by Parental Questionnaire or Recorded Physician Diagnosis (lifetime history)	Recorded Physician Diagnosis verified by ADOS and ADI-R	Asthma: 1.0 (0.69–1.44)
							Results unchanged when considering asthma during pregnancy only
Micali et al. [42]	UK	Case–Control	303 (97/206)	2.5–6.5	Self-Report Questionnaire validated by Medical Records	Clinical Screening then ADI-R	Nonsignificant
Mouridsen et al. [43]	Denmark	Case–control	441 (111/330)	Children with mean age of 5.4	Recorded Physician Diagnosis (ICD-8/10)—lifetime	Recorded Physician Diagnosis (ICD-9)	No association between lifetime history of maternal asthma and autism (p = 0.31)
Patel et al. [18]	Australia	Cohort	363 children with ASD	Children with mean age of 3.07	Self-Report Questionnaire—lifetime	ADOS; Social Responsiveness Scale (measure of severity)	Maternal asthma and allergy condition, more common in male children with ASD comparing to female children with ASD (p = 0.009)

**Table 2:** Studies investigating maternal allergy and autism in offspring

References	Country	Type of study	*N* (case/control)	Age	Method Assessment of Maternal Allergy	Method Assessment of Autism	Study Outcome (95% CI)
Croen et al. [16]	USA	Case–control	2502 (407/2095)	3–7	Recorded Physician Diagnosis (ICD-9) within 2 years of delivery	Recorded Physician Diagnosis (ICD-9)	Allergy: 1.5 (1.2–1.9)
Croen et al. [40]	USA	Case–control	1647 (663/984)	2–5	Computer assisted telephone interview; Prenatal record	SCQ if ≥ 11 then ADOS and ADI-R	Allergy: (0.91–1.41)
Lyall et al. [41]	USA	Case–control	951 (560/391)	2–5	Parental Questionnaire or Recorded Physician Diagnosis (lifetime history and within 2 years of delivery)	Recorded Physician Diagnosis verified by ADOS and ADI-R	All Allergy: 0.97 (0.71–1.32)
							Food 1.23 (0.73–)
							Dairy: 4.31 (1.13–16.5)
							Fruit 1.27 (0.60–2.7)
							Nut 1.42 (0.27–7.64)
							Seafood 2.73 (0.87–8.75)
							Environmental 1.15 (0.82–1.61)
							Medication 0.120 (0.85–1.68)
Patel et al. [18]	Australia	Cohort	363 children with ASD	Children with mean age of 3.07	Self-Report Questionnaire	ADOS; Social Responsiveness Scale (measure of severity)	Maternal asthma and allergy condition, more common in male children with ASD comparing to female children with ASD (p = 0.009)

**Table 3:** Studies investigating maternal eczema and autism in offspring

References	Country	Type of study	*N* (case/control)	Age	Method assessment of maternal eczema	Method assessment of autism	Study outcome (95% CI)
Croen et al. [16]	USA	Case–control	2502 (407/2095)	3–7	Recorded Physician Diagnosis (ICD-9) within 2 years of delivery	Recorded Physician Diagnosis (ICD-9)	Eczema: 1.8 (1.0–3.4)
							Previous History: 1.5 (1.1–2.1)
							Trimester 1: 1.0 (0.4–2.3)
							Trimester 2: 2.5 (1.2–5.2)
							Trimester 3: 1.9 (0.7–4.9)
							Postnatal: 1.5 (1.1–2.0)
Croen et al. [40]	USA	Case–control	1647 (663/984)	2–5	Computer assisted telephone interview; Prenatal record	SCQ if ≥ 11 then ADOS and ADI-R	Eczema and Psoriasis: 1.39 (1.00–1.95)
Micali et al. [42]	UK	Case–control	303 (79/61)	2.5–6.5	Self-Report Questionnaire validated by Medical Records	Clinical Screening then ADI-R	Lifetime History of Eczema: Nonsignificant
Lyall et al. [41]	USA	Case–control	951 (560/391)	2–5	Parental Questionnaire or Recorded Physician Diagnosis (lifetime history and within 2 years of delivery)	Recorded Physician Diagnosis verified by ADOS and ADI-R	Eczema: 1.43 (0.84–2.46)

**Table 4:** Quality assessment of studies using the Newcastle–Ottawa Scale

References	Selection (****)	Comparability (**)	Outcome/exposure (***)	Total score (out of max. 9)
Croen et al. [16]	**	**	**	6
Croen et al. [40]	***	**	*	6
Hisle-Gorman et al. [38]	**	**	**	6
Langridge et al. [39]	**	–	**	4
Leonard et al. [44]	**	**	**	6
Lyall et al. [41]	***	**	**	7
Micali et al. [42]	**	–	*	3
Mouridsen et al. [43]	**	**	**	6
Patel et al. [18]	*	–	**	3

### Asthma

Contradictory findings were noted regarding the association between maternal asthma and autism in offspring (see Table 1) Three of the nine studies demonstrated a significant association, with odds ratios of 1.14 and 1.6, and the cohort study concluding that maternal asthma and allergies are linked with more severe social impairments in the offspring as measured by the Social Responsiveness Scale [16, 18, 38]. Meanwhile, two additional studies showed elevated associations, which, however, became non-significant once adjustment for sociodemographic factors was done [39, 40]. The remaining four studies found evidence of no significant association between the two variables [41–44].

The association between maternal asthma and offspring autism was more consistently significant if it was either treated [38, 40] or recorded during pregnancy, particularly in the first and second trimesters [16], with odds ratios ranging from 1.29 to 2.8. However, this latter finding was not replicated in the secondary analysis of another study [41].

To assess for autism, three of the more recent studies utilized gold-standard assessment tools such as the Autism Diagnostic Observation Schedule (ADOS) and the Autism Diagnostic Interview—Revised (ADI-R), with two of them finding no significant association [40, 41], and one a significant association [18]. The remaining studies ascertained the presence of a diagnosis of autism from medical records; therefore, it is notable that diagnosis in these cases was established via clinical assessment utilizing a method popular in the study location at the time.

Regarding the assessment of maternal asthma, five studies used medical records [16, 38, 39, 43, 44] and two used self-report measures [18, 42]. Two studies categorized mothers as having asthma if the condition was noted in either the medical record or a self-report measure [40, 41].

### Allergy

Regarding maternal allergy and autism, two studies reported positive association [16, 18], with one reporting a notably high odds ratio of 2.5 for allergies diagnosed in the second trimester [16], whereas two studies found no evidence for an association [40, 41] (see Table 2).

Variation was noted in how exposures were assessed: by self-report [18], through maternal records [16], or either[40, 41]. Moreover, studies had varying definitions of what constituted an allergy in terms of exposures and manifestations, for instance two studies categorized eczema as a subtype of allergy [16, 41].

### Eczema

For the relationship between maternal eczema and autism, two studies reported positive association [16, 41], with odds ratios at 1.8 and 1.4, respectively. Meanwhile another study found no evidence for association [42] (see Table 3). Studies again varied as to how exposures were assessed: by self-report [42], through maternal records [16], or either [40, 41]. Discrepancies were noted regarding classification of eczema within atopic and alongside other conditions; for example, eczema was considered as a subtype of allergy in one study [16], while another grouped eczema and psoriasis (an autoimmune condition) together [40].

## Discussion

### Summary of findings

**Fig. 2:**
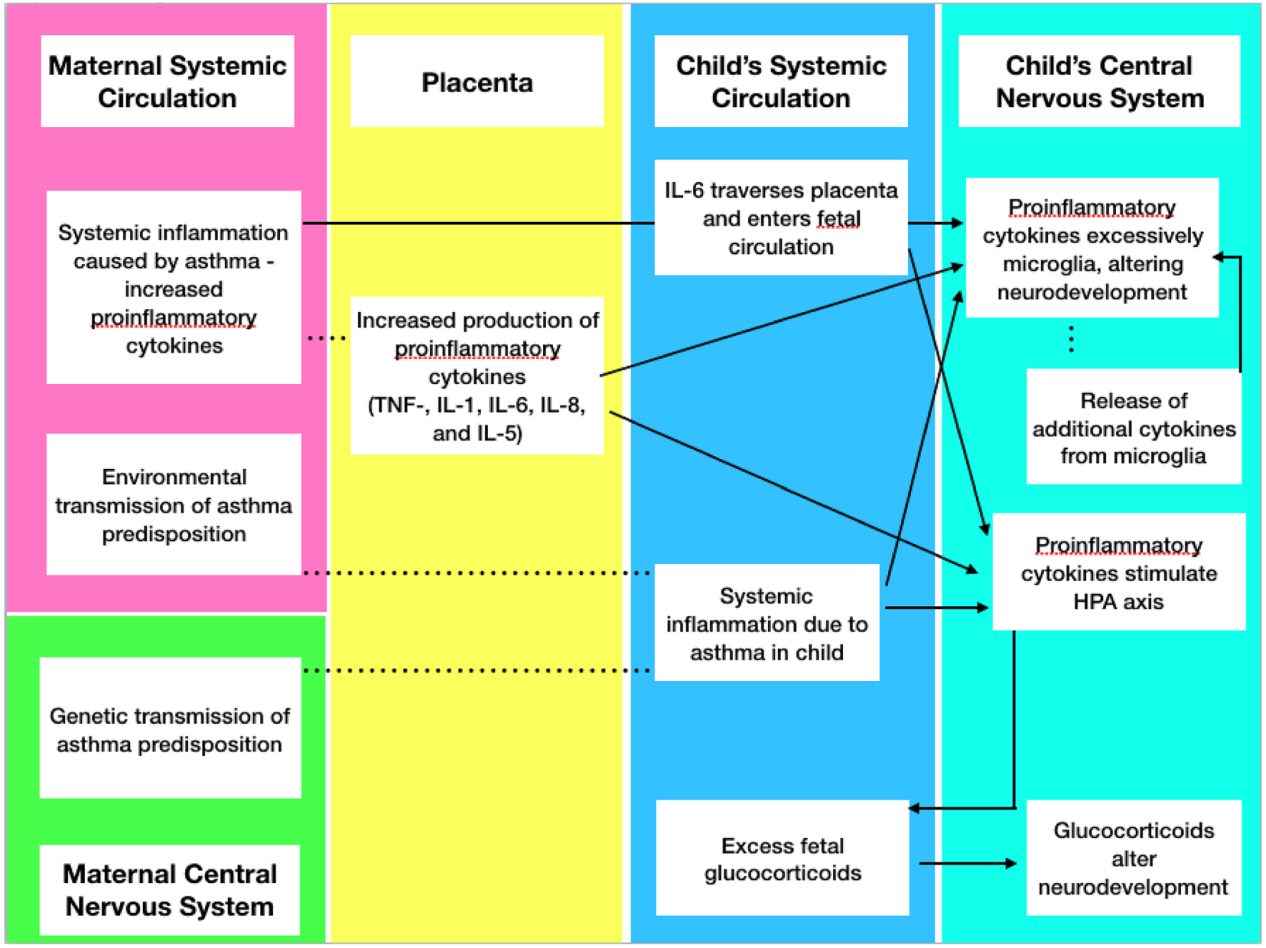
Hypothetical pathways for risk of maternal asthma for autism

In summary, mixed evidence was found regarding the association of a maternal asthma, allergies, or eczema diagnosis with autism in offspring. More consistent associations were demonstrated for asthma that was treated or diagnosed during pregnancy [16, 38, 40], which supports the hypothesis of maternal immune activation. Although most research has focused on autoimmune and infectious triggers, it has been proposed that other conditions such as maternal stress, environmental pollutants, as well as allergies and asthma can also contribute to the risk for ASD by activating maternal immune response [9, 20]. Recent preclinical experiments have demonstrated that a maternal asthma-allergy model is causative of autism-related behaviors in mouse offspring [45, 46]; and cytokines, particularly interleukin (IL)-6, have been proposed to be key mediators of this relationship [47] (see Fig. 2).

Asthma, like many other inflammatory diseases, increases interleukin (IL)-6 levels [48], which is the only proinflammatory cytokine known to pass from maternal to fetal circulation via the placenta [19, 49, 50]. Alternatively, the placenta itself can cause overproduction of IL-6 and other proinflammatory cytokines, a finding demonstrated in preclinical studies and human female offspring of mothers with maternal asthma [19, 51, 52]. Regardless of source, the cytokine imbalance in fetal circulation can breach the immature blood–brain barrier and prime previously quiescent microglia, the resident immune cells of the CNS. Microglia can then transform into a persistently amoeboid morphology, which are both actively phagocytic and capable of producing more cytokines and chemokines, thus causing the neuroinflammatory and neuro-morphological changes seen in ASD [8, 19]. Such a hypothesis is further supported by studies demonstrating increased cytokine levels in midgestational serum of mothers of offspring with ASD [53], as well as in amniotic fluid [54], and newborn blood spots of children eventually diagnosed with ASD [55], although levels of specific cytokines vary in each experiment.

Proinflammatory cytokines can also influence the fetal hypothalamic–pituitary–adrenal (HPA) axis. Whether maternal, placental, or fetal in origin, cytokines can stimulate release of corticotropin releasing hormone and arginine vasopressin from the fetal hypothalamus leading to excess fetal glucocorticoids, which can also affect fetal neurodevelopment and cause long-term changes in function of HPA axis [19, 56–60].

Besides the inhibitory effects of glucocorticoids in fetal neurodevelopment, the use of beta-2adrenergic agonists during pregnancy is found to correlate with greater risk of autism in offspring, independent of maternal asthma [61].

Epigenetic factors have been suggested as a possible mechanism linking maternal allergic asthma and ASD in offspring according to the recent reviews [25, 62].

One of the recent molecular studies also highlights MIA as a well-known risk factor for ASD; it postulates that MIA plays a role at very early stages of fetal neurodevelopment through causing dysregulation of the fetal brain transcriptome and downregulating expression of genes known to be important for ASD pathophysiology [24]. The first genome-wide analyses also point to maternal allergic asthma as a risk factor for ASD through pathogenic pathways that involve modifications in DNA methylation and transcription in microglia isolated from juvenile offspring thus, which impacts on microglial activity which is suggested to be a potential therapeutic target [24].

Maternal inflammation during pregnancy seems to play an important role in immune activation through the placenta and immature blood–brain barrier predisposing the offspring to be susceptible to future hits through microglial activation and modification of fetal epigenetic machinery [25, 62].

Chronic inflammation resulting from an atopic condition could then result in the neuroinflammatory processes described above, resulting in autism [32].

### Study limitations

Some limitations of the studies may have contributed to the inconsistencies in findings. First, regarding the definition of autism, the use of clinical diagnosis to define autism has been subject to questions regarding interrater reliability [63]. Only recently have validated and reliable assessment tools such as the ADOS and ADI-R been used [64], notably accounting for only a third of the studies [18, 40, 41]. Second, the studies included in this review varied in terms of how atopic conditions were defined, diagnosed, and classified. Studies that extracted maternal atopy from medical records were heterogeneous in terms of timeframe, that is whether the diagnosis referred to a lifetime history of asthma, a new diagnosis during pregnancy, or an exacerbation of previously diagnosed asthma during the prenatal period [16, 38, 40, 41, 43, 44]. Variations in the type and classification of allergies and atopic dermatitis also make it challenging to compare studies side-by-side [16, 40, 41]. Third, studies that used self-report to define cases of atopy [18, 40–42] may have been affected by recall bias as parents of children with severe childhood diseases have been known to overreport possible risk factors in an effort to understand the etiology of their child’s condition [65]. Also, none of the studies were adjusted for a family history of autism, to account for genetic liability as a risk factor. Finally, all but one of the studies used a case–control design, which does not allow for a prospective follow-up of maternal asthma and child development, and therefore makes it challenging to presume causality.

### Review limitations

Several limitations of this review must be considered. First, while a conscious decision was made to include articles only published in peer-reviewed journals to ensure quality of the research, some studies affected by publication bias, may have been potentially excluded. Moreover, all the included studies were conducted in high-income countries, possibly limiting the applicability of the results to low- and middle-income countries. Due to high heterogeneity and the inconsistencies between the reported findings in the included studies, a meta-analysis was not conducted; however, applying a meta-analysis following systematic reviews where appropriate would make valuable contributions to the scientific literature. Finally, the Newcastle–Ottawa Scale, the tool used for the risk-of-bias assessment, still has no definitive quantitative norms as to what score constitutes a high- or low-quality study [36]. Therefore, caution must be exercised in using the scores as a measure of study quality.

## Conclusion and recommendations

In conclusion, this review found inconsistent evidence to determine that a diagnosis of asthma, allergies, or eczema is associated with autism in offspring, while there was more consistent evidence for asthma diagnosed or treated during the pregnancy. Such findings, if replicated by differently designed future studies, underscore, both to mothers-to-be, as well as family and professionals caring for them, the importance of effectively managing atopic maternal conditions during the prenatal period as a means to ensure healthy neurodevelopment. More longitudinal studies are likewise recommended for future research, particularly those that utilize gold-standard assessment tools for autism, clearly define the timing of atopic conditions, and correlate exposures with diagnostic (both laboratory and neuroimaging) and treatment data.
